# Controlling the Fiber Stress Distribution with Variable-Frequency Step Roll for Tunable Spun Yarn Structures

**DOI:** 10.3390/polym15132974

**Published:** 2023-07-07

**Authors:** Zhiyong Peng, Wei Li, Ze Chen, Pinxun Wang, Ziyi Su, Yue Sun, Keshuai Liu, Duo Xu, Weilin Xu

**Affiliations:** 1 State Key Laboratory of New Textile Materials and Advanced Processing Technologies, Wuhan Textile University, Wuhan 430200, China; 2 Hubei Key Laboratory of Digital Textile Equipment, Wuhan Textile University, Wuhan 430200, China; 3 College of Textile and Clothing Engineering, Soochow University, Suzhou 215123, China

**Keywords:** step roll, fiber stress, yarn-forming triangle area, variable structure, siro spinning, sirofil spinning

## Abstract

The dynamic regulation of fiber stress distribution in the yarn-forming triangle area is critical for controlling variable composite yarn structures, including siro and sirofil composite yarns. In this study, comparison analyses of the variable geometric structure and stress distribution during the yarn-forming process, which involves step rolls with asymmetrical fiber control, have been carried out using ring-spinning technology. The geometric analyses show that partly staple fibers are continuously controlled while other fibers intermittently lack stress restraint, resulting in cyclically changed helical angles and wrapping density in the yarn-forming triangle area. The yarn structure model displayed that periodically distributed staple fibers occur in siro composite yarn, while sirofil composite yarn shows gradual periodic changes with uniform thickness variations, caused by cyclical changes in the stress distribution between filaments, and the strand altered the yarn-forming zone shapes from symmetrical to offset. Then, a systematic comparison of variable composite yarns with different frequencies (high, medium and low frequency) revealed that low-frequency step roll with wider grooves resulted in an intermittent output of staple fibers with less stress restraint, resulting in more pronounced structural variation in the siro and sirofil composite yarns with a slight yarn quality deterioration.

## 1. Introduction

During ring spinning, the fiber strands form a yarn-forming triangle area after the output from the front nip line, and transfer internally and externally under twist transmission to form the yarn [1,2,3]. Importantly, effective control of the fiber stress in the yarn-forming triangle area is the key to regulating yarn structure. [4,5,6]. However, numerous studies have been conducted with the objective of regulating the stress in the yarn-forming triangle area to reach equilibrium without investigating the effect of differential stress distribution on structure control in yarn formation [7,8], which to some extent limits the development of yarns with controlled variation.

Novel spinning methods [9,10,11,12], such as compact spinning, Nu-torque spinning and groove spinning, have been developed to regulate the fiber stress distribution during yarn formation, thereby adjusting yarn structure as well as improving yarn quality. In compact spinning, a fiber control device is installed in front of the yarn-forming triangle area, which applies a balanced stress on the fiber strands to reduce their width, controlled by airflow, resulting in a ring-spun yarn with tight, clean surface structure and high breaking strength [13,14]. Moreover, groove spinning uses the gradient-changed linear speed of the non-uniform contact surface to balance the fiber stress on both sides of the yarn-forming triangle area, which enables the inter-fibers to be fully transferred with a tighter surface structure and better hairiness and tensile properties [15]. These spinning methods regulate the fiber stress on both sides of the ring-spinning triangle, improving the yarn structure and performance by decreasing the stress difference on the edge fibers [16,17]. However, the above-mentioned spinning methods lack a dynamic regulation of the inter-fiber stresses to obtain yarns with unique transformation structures.

Currently, state-of-the-art studies have been conducted to dynamically regulate the stress distribution between filament and staple fibers, which can obtain variably structured composite yarns [18,19,20]. These composite yarns, with the appearance of cyclic filament embedding and exposure to the yarn body, are controlled by periodically changing the relative positions of staple fibers and filaments [21,22]. Nevertheless, the stress variation in the yarn-forming triangle area is obtained from the dynamic transverse action of the filaments. So far, spinning methods that result in composite yarns with variable structures by cyclically regulating the staple fibers’ stress distribution in the yarn-forming triangle area have not been investigated.

In this study, the morphology of the yarn-forming triangle area was regulated by intermittently expanding the stress difference between staple fibers to produce variable-structure composite yarns. Specifically, step rolls with grooves were used to dynamically regulate the fiber stress distribution at the front nip line, intermittently regulating the wrapping density to form variable-structure siro and sirofil yarns with different fancy effects. The fiber stress distribution and the yarn-forming triangle area’s structure were analyzed with varied periodic frequencies and groove positions of the step rolls. Finally, the different composite yarn structures and performances were tested and compared.

## 2. Theoretical Analysis

### 2.1. Geometric Analysis of the Frequency and Groove Position of the Step Roll Affecting the Fiber Stress Distribution

**Figure 1:**
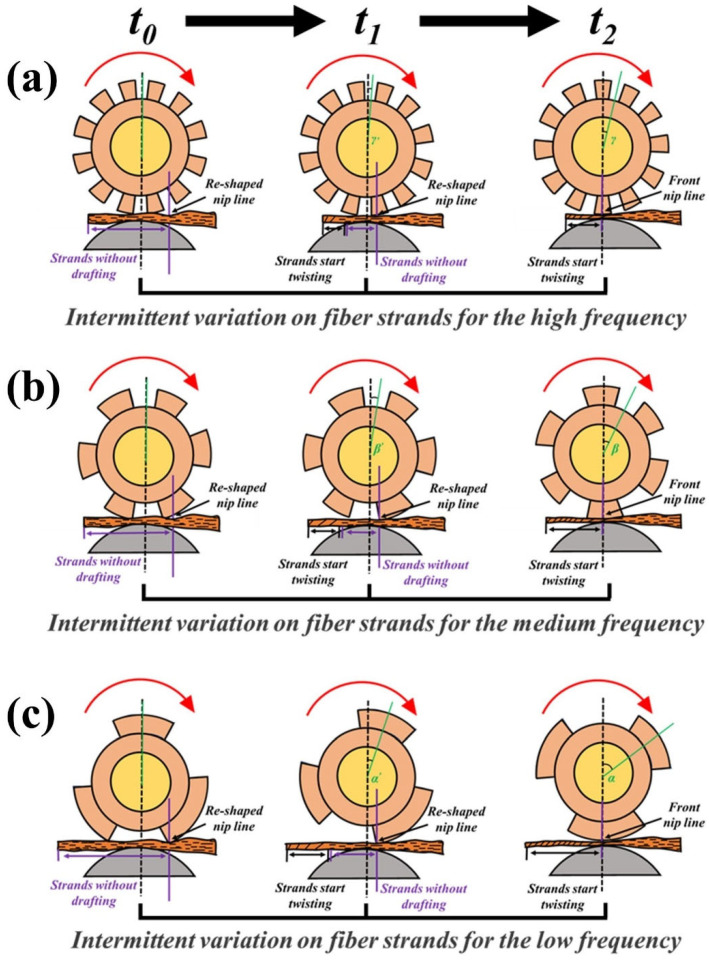
Schematic diagram of intermittently exerted stress on staple fiber strands at different frequencies of step rolls: intermittent variation of stress on fiber strands for the (**a**) high frequency, (**b**) medium frequency and (**c**) low frequency.

Figure 1 illustrates the intermittent gripping of staple fiber strands at different frequencies of step rolls (high, medium and low frequencies). The stress control state produced by the step roll on the staple fiber strands during the yarn-formation process can be divided into three phases (t_0_, t_1_, t_2_). In these three phases, staple fiber strands or filaments on the normal roll side are always subjected to the stresses exerted by the rolls. The three phases correspond to the three states at which the staple fiber strands on the step roll side are not subjected to the stress action at all, are partially subjected to the stress effect and are fully subjected to the stress effect. Different frequencies of step rolls differ in the continuity of stress exerted on the staple fiber strands. The lower the frequency of the step roll, the longer the length of the strands continuously subjected and not subjected to the stress effect, which causes differential fiber stress distribution for variable structure control.

### 2.2. Geometric Analysis of the Groove Position for the Step Roll Affecting the Shape of the Yarn-Forming Triangle Area and Fiber Wrapping Form

**Figure 2:**
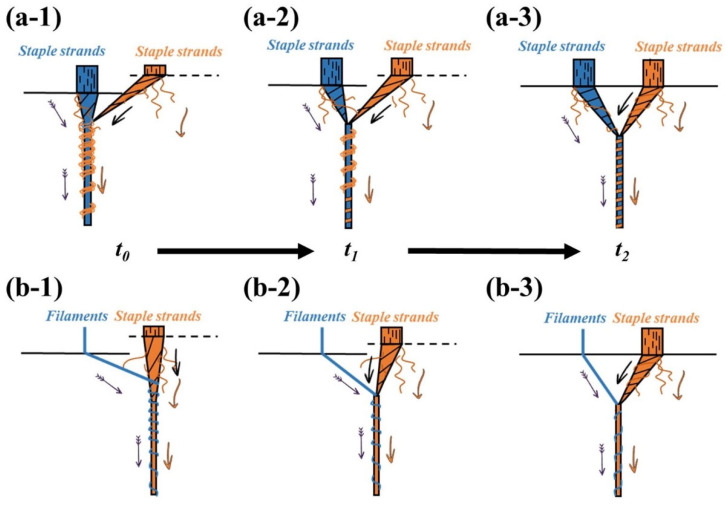
Geometric analysis of the groove position of the step roll affecting the shape of yarn-forming triangle area and fiber wrapping form: (**a-1**) siro spinning at stage t_0_; (**a-2**) siro spinning at stage t_1_; (**a-3**) siro spinning at stage t_2_; (**b-1**) sirofil spinning at stage t_0_; (**b-2**) sirofil spinning at stage t_1_; (**b-3**) sirofil spinning at stage t_2_.

**Figure 3:**
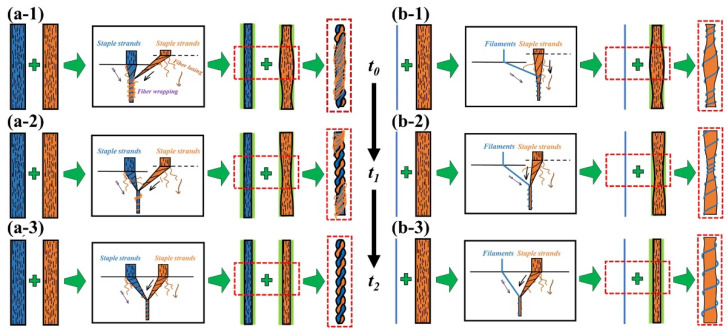
Model of effect of step roll groove position on yarn count and unevenness: (**a-1**) siro spinning at stage t_0_; (**a-2**) siro spinning at stage t_1_; (**a-3**) siro spinning at stage t_2_; (**b-1**) sirofil spinning at stage t_0_; (**b-2**) sirofil spinning at stage t_1_; (**b-3**) sirofil spinning at stage t_2_.

During ring spinning, variations in the yarn-forming triangle area’s morphology largely influence the performance of yarn formation [23,24,25]. Figure 2 demonstrates the change in fiber movement in the yarn-forming triangle area during the spinning of the siro as well as the sirofil. For siro composite yarn, while the staple fiber strands on the step roll side sit inside the groove (Figure 2(a-1,a-2)), the strands remain loose and under stress, and get pulled to the normal roll side to form an oblique yarn-forming triangle area due to the staple fiber strands on the normal roll side being under a fully stressed state. Then, the stacked fiber bundles output from the step roll side become twisted and combine with the staple fiber strands on the normal roll side at a larger wrapping angle, forming a more densely wrapped and thicker yarn segment. In addition, the oblique yarn-forming triangle area causes some fiber loss because the staple fiber strands suffer from oblique pulling, which increases their area without stress. As the step roll rotates, its groove edge starts to come into contact with the staple fiber strands and exerts stress. Once the re-shaped nip line coincides with the front nip line (Figure 1), the yarn-forming triangle area exhibits a symmetrical distribution (Figure 2(a-3,b-3)) due to the step roll’s stress effect on the staple fiber strands being same as that of the normal roll, allowing both bundles of staple fiber strands to exit from the front nip at the same speed.

For sirofil composite yarn, the yarn-forming triangle area intermittently leans to the step roll side (Figure 2(b-1,b-2)), mainly because of the heavier weight of staple fiber strands relative to the filament, which draws the filament toward itself. Remarkably, as the filament gradually leans toward the strands, the angle and density of the filament wrapping around the staple fiber strands increase, as well as the binding force. Therefore, the staple fibers at the edge of the strands are captured by the filament and embedded in the backbone of the yarn, which improves the hairiness of the composite yarn to some extent.

### 2.3. The Model of the Effect of Step Roll Frequency and Groove Position on Yarn Structure

According to the geometric analysis, the siro spinning system with two strands of staple fiber strands lacks the ability to control the edge staple fibers (Figure 3(a-1,a-2)), which tends to cause a certain degree of fiber losses, reducing the yarn count. On the contrary, the sirofil spinning system has a superior ability to capture staple fibers (Figure 3(b-1,b-2)), so even fallen fibers are captured by the filament and embedded in the backbone of the yarn, thus reducing staple fiber loss to a large extent and ensuring yarn count stability.

**Figure 4:**
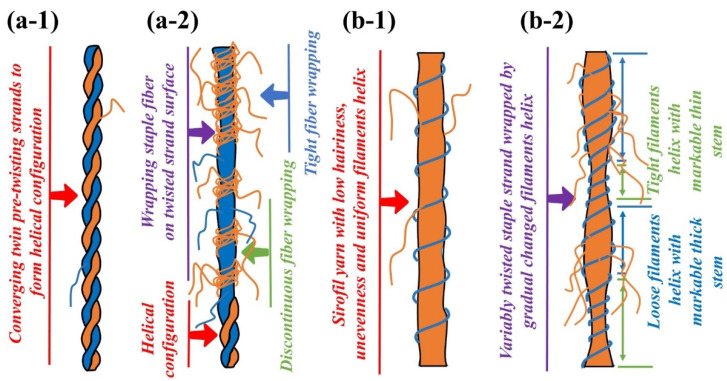
Model of effect of fiber wrapping on structural morphology of yarn formation: (**a-1**) converging twin pre-twisting strands to form helical configuration; (**a-2**) wrapping staple fiber with uneven distribution on twisted strand surface; (**b-1**) sirofil yarn with low hairiness, unevenness and uniform filament helix; (**b-2**) variably twisted staple strand wrapped by gradually changing filament helix.

In the t_0_ stage (Figure 3(a-1,b-1)), the staple fiber strands on the step roll side are without stress and completely relaxed, and the main part of the thicker yarn sections is formed when the strands are output from the front nip line (Figure 4(a-2,b-2)). At stage t_1_ (Figure 3(a-2,b-2)), as the staple fiber strands are under less stress and partially relax on the side of the step roll, the output strands from the front nip line end up in a gradual thickness of yarn (Figure 4(a-2,b-2)). When leaving t_1_ and reaching t_2_ (Figure 3(a-3,b-3)), the staple fibers are always subjected to larger stress in the gripping state, and the output speed is consistent with the normal roll on the other side, resulting in a normal thickness and spiral structure of the siro spun and sirofil spun yarns (Figure 4(a-1,b-1)).

## 3. Materials and Methods

### 3.1. Materials

The raw materials used in this experiment were cotton fiber, viscose fiber and polyester filament. In order to demonstrate the above theory of variable-structure composite yarn generation by groove step rolls, firstly, step rolls were installed on a Dssp-01 ring-spinning machine and two strands of pure cotton and viscose fiber, both 700 tex, were used to produce the siro and sirofil variable-structure composite yarns. The detailed process parameters are presented in Table 1.

### 3.2. Experimental Details

Two strands were fed from the bell and then passed through the general drafting area, the front nip line, the yarn guide hook, the wire loop and the spindle in turn. Notably, in the case of compounding staple fibers and filaments, the filaments did not pass through the drafting zone, but through the tension disk, and then through the yarn guide wheel and out through the front nip line together with the staple fiber strands.

**Figure 5:**
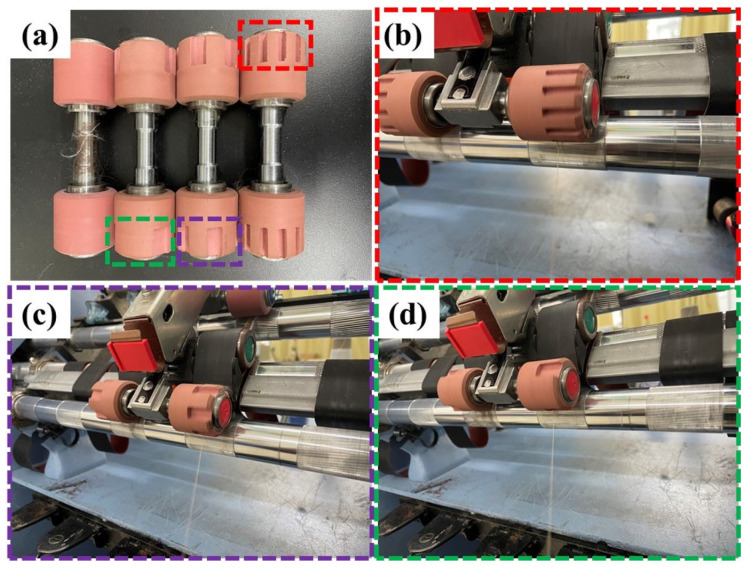
Optical image of different frequency step rolls and production of variable-structure composite yarns on a ring-spinning frame: (**a**) normal roll and three types of step rolls; (**b**) high-frequency step roll on the machine; (**c**) medium-frequency step roll on the machine; (**d**) low-frequency step roll on the machine.

In order to better compare the quality of yarn spun under different groove step rolls, three different frequencies of step roll (low frequency, medium frequency and high frequency) were used for comparison with the normal roll. From Figure 5, it can be clearly observed that the step roll is divided into two parts, one half of which is the normal roll and the other half is the groove roll, and the step roll is also installed above the front roller. In addition, as shown in Table 2, four types of siro yarns (OSY, HSY, MSY, LSY) and four types of sirofil yarns (OSFY, HSFY, MSFY, LSFY) were set up in this experiment, where OSY and OSFY were yarns spun with normal roll and the rest were yarns spun with step roll.

All samples for this experiment were stored for at least 24 h under specific conditions (temperature of 25 ± 2 °C and relative humidity of 65 ± 2%) of standard atmospheric pressure. An iPhone 14 pro max was used to record the dynamic process of the yarn-forming triangle area on the ring-spinning frame. The surface structure of the variable-structure composite yarns was observed using an RH-2000 electron microscope (produced by Japan Hirox Co., Ltd., Tokyo, Japan). According to the CN FZ/T 01086-2020 standard [26], an H400 hairiness tester (produced by China Suzhou Changfeng Co., Ltd., Suzhou, China) was used to test each group of samples at a speed of 30 m/min and the average value was taken from 10 sets of experimental data. An E500 evenness meter (produced by China Suzhou Changfeng Co., Ltd.) was used to test the unevenness and defects of variable-structure composite yarns under the CN GB/T 3292.1-2008 standard [27] and the average value was taken from 10 sets of data. For the tensile strength test, a YG068C single-yarn tensile tester (produced by China Suzhou Changfeng Co., Ltd.) with a length of 500 mm and a speed of 500 mm/min was used for the test (in accordance with the CN GB/T 3916-2013 standard [28]), and the average value was taken after 10 sets of consecutive tests. Furthermore, the Student–Newman–Keuls (SNK) tests were carried out by using SPSS program. The significance level (*p*) of SNK tests was indicated as * in the bar charts (* means a difference with *p* < 0.05 and ** shows a difference with *p* < 0.01).

## 4. Results and Discussion

### 4.1. Dynamic Change in Yarn-Forming Triangle Area

**Figure 6:**
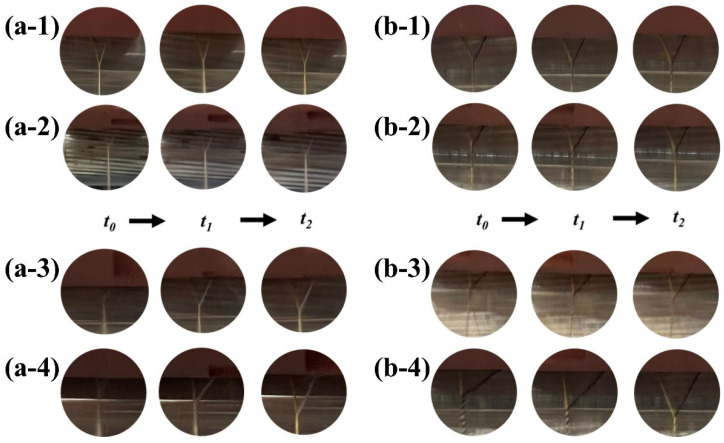
The variation in the yarn-forming triangle area during the actual spinning process: (**a-1**) OSY; (**a-2**) HSY; (**a-3**) MSY; (**a-4**) LSY; (**b-1**) OSFY; (**b-2**) HSFY; (**b-3**) MSFY; (**b-4**) LSFY.

The yarn-forming triangle area is tilted intermittently when the staple fiber or filament enters the front nip line and outputs, resulting in the groove of the step rolls. In addition, the area of the yarn-forming triangle area increases with the increase in the width of the groove of the step rolls (Figure 6). As can be seen, the dynamic migration changes in the yarn-forming triangle area in siro and sirofil variable-structure composite yarns are consistent with the theoretical analysis (Figure 2).

### 4.2. Comparison of Surface Structure of Yarns Spun by Different Step Rolls

**Figure 7:**
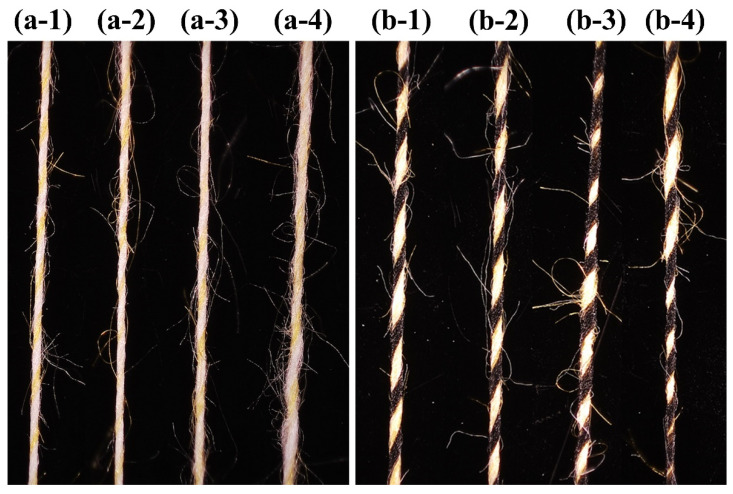
Surface structure characterization of yarns spun by different step rolls: (**a-1**) OSY; (**a-2**) HSY; (**a-3**) MSY; (**a-4**) LSY; (**b-1**) OSFY; (**b-2**) HSFY; (**b-3**) MSFY; (**b-4**) LSFY.

**Figure 8:**
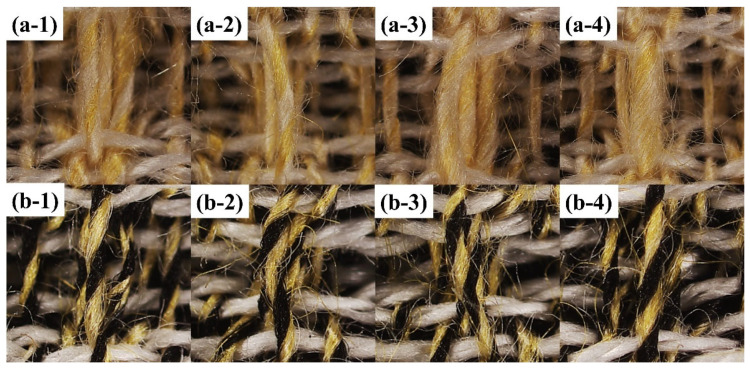
Microscopic image of fabrics: (**a-1**) OSY; (**a-2**) HSY; (**a-3**) MSY; (**a-4**) LSY; (**b-1**) OSFY; (**b-2**) HSFY; (**b-3**) MSFY; (**b-4**) LSFY.

Figure 7 demonstrates the microstructure of two different groups of composite yarns. As can be seen, the control groups OSY and OSFY have uniform evenness, excellent hairiness and tight yarns, mainly due to the two strands being evenly output and fully transferred to each other. However, the rest of the yarns show structures with thickness variations of different degrees, which are mainly determined by the groove frequency of the step roll. The appearance of the structures of HSY and HSFY is extremely similar to the control groups OSY and OSFY, which is mostly caused by the small spacing of the high-frequency step roll, short intervals between intermittent stresses on the staple fibers by the roll and front roller and the strands being brought out again into the normal roll area before they were completely inside the grooves. The most obvious structures with thickness variation can be observed in Figure 7(a-4,b-4) (LSY and LSFY), since the step roll and front roller failed to grip the strands when they passed through the groove, resulting in an accumulation of the strands in the groove area without stress and thus the formation of variable-structure composite yarns. As is evident from Figure 8, the fabrics made of siro and sirofil variable-structure composite yarns still present a fancy effect of varying thickness, which can be applied to curtains, carpets and handicrafts as required.

### 4.3. Comparison of Hairiness of Yarns Spun by Different Step Rolls

**Figure 9:**
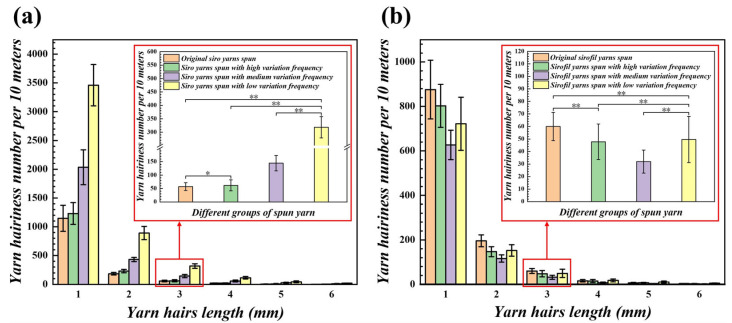
Hairiness number comparison of different variable-structure composite yarns: (**a**) hairiness of the siro yarns; (**b**) hairiness of the sirofil yarns (* means a difference with *p* < 0.05 and ** shows a difference with *p* < 0.01).

The observation in Figure 9 shows that LSY has the most hairiness compared to the remaining three groups of the same kind (*p* < 0.05, Appendix A), as seen in the 3 mm hairiness in the enlarged figure, which is similar in principle to the variable-structure composite yarn generation illustrated above. On the one hand, the groove area of the step roll spun for LSY is the largest, which allows the staple fiber strands to pass through the groove section of the low-frequency step roll for the longest period of time without stress, and the edge staple fibers are unable to embed well into the yarn stem, resulting in a sharp increase in the amount of hairiness. On the other hand, the intermittent movement of the strands in and out of the grooves causes pressure on the edges from the grooves. Under constant pressure, the smaller the contact area is, the greater the pressure, which causes some fiber damage and leads to more hairiness. Noticeably, when filament and staple fibers are composite, the control group OSFY has the most hairiness, which might be due to the staple fiber strands of the control group OSFY being under stress exerted by the front roller and normal roll and twisting with the filaments in the form of parallel and loose states; some staple fibers in the twisting triangle are separated from the yarn stem due to insufficient stress and then form hairiness, even causing fiber loss in the form of fallen fiber. Fiber loss results in a slight decrease in yarn linear density (*p* < 0.05, Appendix B). However, in the other three experimental groups, the strands are intermittently not under stress due to the presence of grooves in the step roll, and the filaments are deflected by the strands in the form of small fiber pieces, so the yarn-forming triangle area is tilted toward the strand side and the wrapping angle and density of the filament on the strands increase. Then, the strands are bound by the filaments, which makes them less likely to break away from the yarn stem and reduces the amount of hairiness.

### 4.4. Comparison of Evenness of Yarns Spun by Different Step Rolls

**Figure 10:**
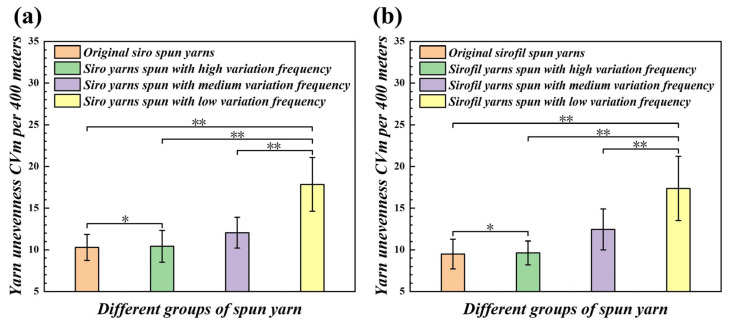
Evenness comparison of different variable-structure composite yarns: (**a**) evenness in stem of the siro yarns; (**b**) evenness in stem of the sirofil yarns (* means a difference with *p* < 0.05 and ** shows a difference with *p* < 0.01).

**Figure 11:**
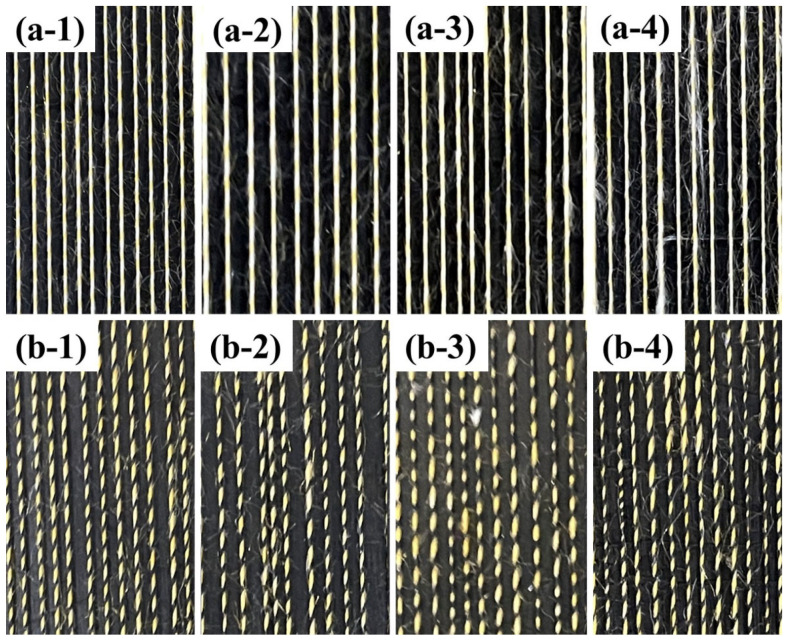
Macroscopic characterization of yarn quality by the blackboard method: (**a-1**) OSY; (**a-2**) HSY; (**a-3**) MSY; (**a-4**) LSY; (**b-1**) OSFY; (**b-2**) HSFY; (**b-3**) MSFY; (**b-4**) LSFY.

Yarn evenness refers to the uniformity of yarn thickness or weight in short sections along the axial direction [29,30]. Evenness is an important index of yarn quality, which not only affects the single-yarn strength and the variation factor of the strength, but also affects the weaving breakage rate and fabric flatness [31,32]. From Figure 10 and Figure 11, it can be seen that in terms of the evenness of the yarns, the general direction is the same for both the two strands of staple fibers and the composite of filaments and staple fiber strands (*p* < 0.05, Appendix C). On the normal roll, the strands are evenly stressed at the front nip line, and the output is carried out normally, producing a more uniform yarn. Nevertheless, when using a step roll, the drafting of the strands is interrupted by the lack of a stress effect in the groove, which results in yarn structures with thickness variations and is also the primary cause of unevenness. In addition, the strands will have some friction and resistance when passing through the edge of groove, which will cause the strands to be easily stretched or deformed and also affect the uniformity of the yarn stem.

### 4.5. Comparison of Tensile Properties of Yarns Spun by Different Rolls

**Figure 12:**
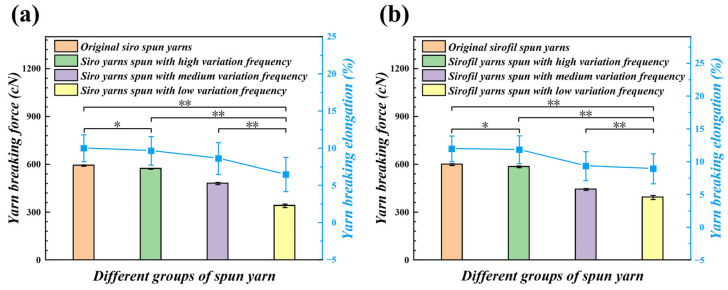
Tensile comparison of different variable-structure composite yarns: (**a**) breaking strength of the siro yarns; (**b**) breaking strength of the sirofil yarns (* means a difference with *p* < 0.05 and ** shows a difference with *p* < 0.01).

Figure 12 reveals the tensile properties of variable-structure composite yarns at different frequencies of the grooved roll. The results show that the breaking strength of yarn passing through the step roll decreases with a reduction in groove frequency (*p* < 0.05, Appendix D). The reason is that the strands in the groove are not under stress exerted by the front roller and the step roll, and the wire loop does not transfer enough twist back to the yarn-forming triangle area, which leads to less twist in the segment as well as lower fiber cohesion, resulting in a decrease in the yarn strength of this segment.

Another key factor may be that the staple fibers passing through the groove area are exposed to the yarn stem, or even some of the fibers are damaged when passing through the groove edge of the step roll, resulting in lower fiber strength utilization. On the contrary, reducing the fiber slippage space by adjusting the fiber arrangement when the yarn passes through the normal roll could make the staple fibers to be fully transferred inside and outside and ensure a uniform force on the fibers in the yarn.

## 5. Conclusions

In this article, it is investigated that variable-structure siro and sirofil composite yarns could be produced on a ring-spinning machine using a variable-frequency step roll. When the yarn is on the normal roll, the strands are found to be drawn and elongated by the large stress effect, and upon entering the interior of the groove, the fiber strands are output directly from the front nip line without stress and draw. Then, the strands merge with the staple fibers or filaments that are output from the normal roll on the other side to form a variable-structure composite yarn. Furthermore, experiments are performed to compare the differences in the hairiness, evenness and tensile properties between the composite yarns at different frequencies of the rolls.

The results demonstrated that the step roll has a significant effect on the physical as well as mechanical properties of the composite yarns. The hairiness, evenness and breaking strength of the variable-structure siro and sirofil composite yarns are slightly inferior compared to the normal siro and sirofil yarns. However, variable-structure composite yarns with fancy effects are significant in both the production and use of sustainable textiles. The unique and attractive textures of variable-structure composite yarns made with a variable-frequency step roll can be used in a wide range of applications, such as home decoration and handicrafts.

## Figures and Tables

**Table 1:** Specific spinning process parameters.

Parameter	Numerical Value
Theoretical spindle speed (r/min)	4500
Theoretical linear density (tex)	19.7
Whole draft ratio	36.2
Twist factor	400
Twist direction	Z
Twist shrinkage (%)	3.5
Front roller speed (m/min)	12.01
Bar spacer (mm)	3.0
Ring type	PG1/2 3854
Traveler type	6903 4/0
Groove width of high-frequency step roll (mm)	3
Groove width of medium-frequency step roll (mm)	6
Groove width of low-frequency step roll (mm)	9

**Table 2:** Experiment design of variable-structure composite yarns.

Yarn Code	Yarn Type
OSY	Original siro spun yarns
HSY	Siro yarns spun with high-variation frequency
MSY	Siro yarns spun with medium-variation frequency
LSY	Siro yarns spun with low-variation frequency
OSFY	Original sirofil spun yarns
HSFY	Sirofil yarns spun with high-variation frequency
MSFY	Sirofil yarns spun with medium-variation frequency
LSFY	Sirofil yarns spun with low-variation frequency

**Figure A1:**
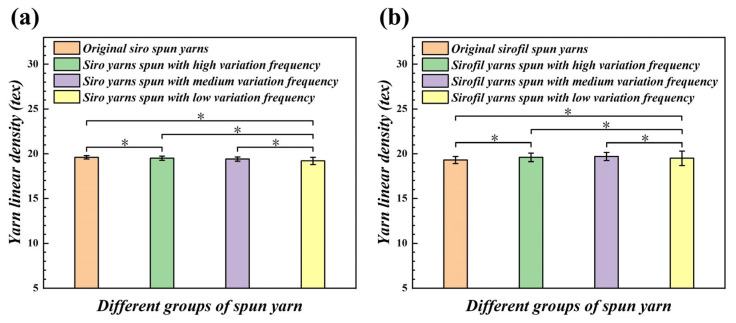
Linear density comparison of different variable structure composite yarns: (**a**) linear density of the siro yarns; (**b**) linear density of the sirofil yarns (* means a difference with *p* < 0.05).

## Data Availability

Not applicable.

## Appendix A. Statistical Test Results for 3 mm Hairiness Index of Different Yarns (Figure 9) at a Significance Level of 0.05 are Shown in Table A1 and Table A2

**Table A1:** 3mm hairiness index comparison for 19.7 tex between original siro spun yarns, siro yarns spun with high variation frequency, siro yarns spun with medium variation frequency, and siro yarns spun with low variation frequency.

Student-Newman-Keulsa ^a^					
		Subset for Alpha = 0.05			
VAR00001	N	1	2	3	4
1. Original siro spun yarns	30	57.2			
2. Siro yarns spun with high variation frequency	30		61.8		
3. Siro yarns spun with medium variation frequency	30			144.6	
4. Siro yarns spun with low variation frequency	30				318.6
Sig.		1.000	1.000	1.000	1.000

Means for groups in homogeneous subsets are displayed. ^a^ Uses harmonic mean sample size = 30.000.

**Table A2:** 3mm hairiness index comparison for 19.7 tex between original sirofil spun yarns, sirofil yarns spun with high variation frequency, sirofil yarns spun with medium variation frequency, and sirofil yarns spun with low variation frequency.

Student-Newman-Keulsa ^a^					
		Subset for Alpha = 0.05			
VAR00001	N	1	2	3	4
1. Original sirofil spun yarns	30	60			
2. Sirofil yarns spun with high variation frequency	30		47.8		
3. Sirofil yarns spun with medium variation frequency	30			32	
4. Sirofil yarns spun with low variation frequency	30				49.6
Sig.		1.000	1.000	1.000	1.000

Means for groups in homogeneous subsets are displayed. ^a^ Uses harmonic mean sample size = 30.000.

## Appendix B. Statistical Test Results for Linear Density of Different Yarns (Figure A1) at a Significance Level of 0.05 are Shown in Table A3 and Table A4

**Table A3:** Linear density comparison for 19.7 tex between original siro spun yarns, siro yarns spun with high variation frequency, siro yarns spun with medium variation frequency, and siro yarns spun with low variation frequency.

Student-Newman-Keulsa ^a^					
		Subset for Alpha = 0.05			
VAR00001	N				1
1. Original siro spun yarns	30				19.6
2. Siro yarns spun with high variation frequency	30				19.5
3. Siro yarns spun with medium variation frequency	30				19.4
4. Siro yarns spun with low variation frequency	30				19.2
Sig.					0.113

Means for groups in homogeneous subsets are displayed. ^a^ Uses harmonic mean sample size = 30.000.

**Table A4:** Linear density comparison for 19.7 tex between original sirofil spun yarns, sirofil yarns spun with high variation frequency, sirofil yarns spun with medium variation frequency, and sirofil yarns spun with low variation frequency.

Student-Newman-Keulsa ^a^					
		Subset for Alpha = 0.05			
VAR00001	N	1	2	3	1
1. Original sirofil spun yarns	30				19.3
2. Sirofil yarns spun with high variation frequency	30				19.6
3. Sirofil yarns spun with medium variation frequency	30				19.7
4. Sirofil yarns spun with low variation frequency	30				19.5
Sig.					0.260

Means for groups in homogeneous subsets are displayed. ^a^ Uses harmonic mean sample size = 30.000.

## Appendix C. Statistical Test Results for Irregularities of Different Yarns (Figure 10) at a Significance Level of 0.05 are Shown in Table A5 and Table A6

**Table A5:** Unevenness CVm comparison for 19.7 tex between original siro spun yarns, siro yarns spun with high variation frequency, siro yarns spun with medium variation frequency, and siro yarns spun with low variation frequency.

Student-Newman-Keulsa ^a^					
		Subset for Alpha = 0.05			
VAR00001	N	1	2	3	4
1. Original siro spun yarns	30	10.29			
2. Siro yarns spun with high variation frequency	30		10.43		
3. Siro yarns spun with medium variation frequency	30			12.05	
4. Siro yarns spun with low variation frequency	30				17.85
Sig.		1.000	1.000	1.000	1.000

Means for groups in homogeneous subsets are displayed. ^a^ Uses harmonic mean sample size = 30.000.

**Table A6:** Unevenness CVm comparison for 19.7 tex between original sirofil spun yarns, sirofil yarns spun with high variation frequency, sirofil yarns spun with medium variation frequency, and sirofil yarns spun with low variation frequency.

Student-Newman-Keulsa ^a^					
		Subset for Alpha = 0.05			
VAR00001	N	1	2	3	4
1. Original sirofil spun yarns	30	9.49			
2. Sirofil yarns spun with high variation frequency	30		9.63		
3. Sirofil yarns spun with medium variation frequency	30			12.46	
4. Sirofil yarns spun with low variation frequency	30				17.36
Sig.		1.000	1.000	1.000	1.000

Means for groups in homogeneous subsets are displayed. ^a^ Uses harmonic mean sample size = 30.000.

## Appendix D. Statistical Test Results for Tensile Properties of Different Yarns (Figure 12) at a Significance Level of 0.05 are Shown in Table A7 and Table A8

**Table A7:** Breaking force comparison for 19.7 tex between original siro spun yarns, siro yarns spun with high variation frequency, siro yarns spun with medium variation frequency, and siro yarns spun with low variation frequency.

Student-Newman-Keulsa ^a^					
		Subset for Alpha = 0.05			
VAR00001	N	1	2	3	4
1. Original siro spun yarns	30	594.33			
2. Siro yarns spun with high variation frequency	30		574.94		
3. Siro yarns spun with medium variation frequency	30			482.01	
4. Siro yarns spun with low variation frequency	30				342.27
Sig.		1.000	1.000	1.000	1.000

Means for groups in homogeneous subsets are displayed. ^a^ Uses harmonic mean sample size = 30.000.

**Table A8:** Breaking force comparison for 19.7 tex between original sirofil spun yarns, sirofil yarns spun with high variation frequency, sirofil yarns spun with medium variation frequency, and sirofil yarns spun with low variation frequency.

Student-Newman-Keulsa ^a^					
		Subset for Alpha = 0.05			
VAR00001	N	1	2	3	4
1. Original sirofil spun yarns	30	600.77			
2. Sirofil yarns spun with high variation frequency	30		586.4		
3. Sirofil yarns spun with medium variation frequency	30			444.53	
4. Sirofil yarns spun with low variation frequency	30				394.76
Sig.		1.000	1.000	1.000	1.000

Means for groups in homogeneous subsets are displayed. ^a^ Uses harmonic mean sample size = 30.000.
